# Reply to Su et al. Comment on “Matsumoto et al. Remimazolam’s Effects on Postoperative Nausea and Vomiting Are Similar to Those of Propofol after Laparoscopic Gynecological Surgery: A Randomized Controlled Trial. *J. Clin. Med.* 2023, *12*, 5402”

**DOI:** 10.3390/jcm13041002

**Published:** 2024-02-09

**Authors:** Ayumu Matsumoto, Shiho Satomi, Nami Kakuta, Soshi Narasaki, Yukari Toyota, Hirotsugu Miyoshi, Yousuke T. Horikawa, Noboru Saeki, Katsuya Tanaka, Yasuo M. Tsutsumi

**Affiliations:** 1 Department of Anesthesiology and Critical Care, Hiroshima University, Hiroshima 734-8553, Japan; 2 Department of Anesthesiology, Tokushima University, Tokushima 770-8503, Japan

We thank the authors for their insightful and thoughtful commentary on our recent publication [1]. We calculated the sample size based on the data comparing the incidence of PONV between sevoflurane and propofol because remimazolam is a new intravenous anesthetic approved for the first time in the world in Japan in 2020 for general anesthesia and we could not find data on PONV caused by total intravenous anesthesia except for propofol at that time. We revealed the incidence of PONV caused by remimazolam compared to propofol, and the odds ratio was 1. This result suggests there was no difference in the incidence of PONV between remimazolam and propofol. Therefore, we believe that our small sample size contributed very little to the result indicating there was no significant difference in the incidence of PONV in this study.

According to guidelines for the management of PONV, perioperative fasting has an uncertain significance, and the occurrence of intraoperative hypotension was not included in the risk factors for PONV [2]. We defined intraoperative hypotension to set criteria for the administration of ephedrine during general anesthesia.

For postoperative analgesia, non-opioid analgesics were also used. In addition, pentazocine and/or buprenorphine were administered if necessary. There were no significant differences between the remimazolam and propofol groups in the use of pentazocine, buprenorphine, and remifentanil in this study.
